# Biomarkers of Vitamin D Metabolism and Hip and Vertebral Fracture Risk: The Multi‐Ethnic Study of Atherosclerosis

**DOI:** 10.1002/jbm4.10697

**Published:** 2022-11-11

**Authors:** Simon Hsu, Michael H. Criqui, Charles Ginsberg, Andrew N. Hoofnagle, Joachim H. Ix, Robyn L. McClelland, Erin D. Michos, Steven J. Shea, David Siscovick, Leila R. Zelnick, Bryan R. Kestenbaum, Ian H. de Boer

**Affiliations:** ^1^ Division of Nephrology and Kidney Research Institute, Department of Medicine University of Washington Seattle WA USA; ^2^ Division of Preventive Medicine, Department of Family Medicine University of California, San Diego La Jolla CA USA; ^3^ Division of Nephrology‐Hypertension University of California, San Diego San Diego CA USA; ^4^ Department of Laboratory Medicine University of Washington Seattle WA USA; ^5^ Department of Biostatistics University of Washington Seattle WA USA; ^6^ Division of Cardiology, Department of Medicine Johns Hopkins University Baltimore MD USA; ^7^ Department of Epidemiology and the Welch Center for Prevention, Epidemiology and Clinical Research Johns Hopkins University Bloomberg School of Public Health Baltimore MD USA; ^8^ Department of Medicine Columbia University College of Physicians and Surgeons New York NY USA; ^9^ Department of Epidemiology Mailman School of Public Health, Columbia University New York NY USA; ^10^ New York Academy of Medicine New York NY USA

**Keywords:** FRACTURE PREVENTION, FRACTURE RISK ASSESSMENT, PTH/VITAMIN D/FGF23

## Abstract

Studies on associations between biomarkers of vitamin D metabolism and fracture risk have focused predominantly on White or elderly populations and may not be generalizable to relatively healthy multiethnic populations. We tested associations of total 25‐hydroxyvitamin D (25[OH]D), the ratio of 24,25‐dihydroxyvitamin D_3_ to 25‐hydroxyvitamin D_3_ (vitamin D metabolite ratio, VDMR), parathyroid hormone (PTH), and fibroblast growth factor‐23 (FGF‐23) concentrations measured in serum with risk of hip and vertebral fractures in the Multi‐Ethnic Study of Atherosclerosis (MESA). Serum 25‐hydroxyvitamin D_2_ and D_3_ and 24,25‐dihydroxyvitamin D_3_ were measured by liquid chromatography–tandem mass spectrometry (LC–MS/MS). The study cohort of 6466 participants was without clinically apparent cardiovascular disease and was 39% White, 27% Black, 22% Hispanic, and 12% Chinese. The mean age was 62 years, and 53% were female. There were 128 hip and vertebral fractures over a mean follow‐up of 14.2 years. 25(OH)D, the VDMR, PTH, and FGF‐23 were not significantly associated with fracture risk after adjustment for demographics, diabetes, smoking, systolic blood pressure, body mass index, medication use, albuminuria, and estimated glomerular filtration rate. Principal component analysis did not suggest differences in linear combinations of 25(OH)D, the VDMR, PTH, and FGF‐23 between participants who experienced fractures and those who did not. We did not observe significant interaction between race and ethnicity and any biomarker of vitamin D metabolism on fracture risk. In conclusion, none of the four serum biomarkers of vitamin D metabolism investigated showed a significant association with fracture risk in relatively healthy multiethnic populations. © 2022 The Authors. *JBMR Plus* published by Wiley Periodicals LLC on behalf of American Society for Bone and Mineral Research.

## Introduction

Vitamin D is an essential nutrient for bone mineralization and fracture prevention.^(^ ^1^, ^2^ ^)^ Thus, there is an extensive literature examining whether biomarkers of vitamin D metabolism can be used as tools to assess fracture risk. The circulating concentration of 25‐hydroxyvitamin D (25[OH]D) is most widely used to define vitamin D status, yet it is an inactive vitamin D metabolite inconsistently associated with bone mineral density (BMD) and fractures.^(^ ^3^, ^4^, ^5^, ^6^, ^7^ ^)^ Alternative biomarkers that reflect functional vitamin D activity have seen increased interest in their potential role in fracture risk assessment. Vitamin D receptor activation by 1,25‐dihydroxyvitamin D (1,25[OH]_2_D, the biologically active form of vitamin D) induces the metabolism of 25(OH)D into 24,25‐dihydroxyvitamin D (24,25[OH]_2_D).^(^ ^8^, ^9^ ^)^ Consequently, the ratio of 24,25(OH)_2_D_3_ to 25(OH)D_3_ (vitamin D metabolite ratio, VDMR) has been used as a readout of tissue‐level vitamin D activity, and our group has shown lower VDMR is associated with fracture risk in older, high‐risk cohorts.^(^ ^3^, ^4^ ^)^ Similarly, 1,25(OH)_2_D directly regulates the synthesis and secretion of parathyroid hormone (PTH) and fibroblast growth factor‐23 (FGF‐23), which in turn reciprocally modulate the conversion of 25(OH)D into 1,25(OH)_2_D.^(^ ^10^, ^11^, ^12^ ^)^ As such, studies have also reported on associations of PTH and FGF‐23 with bone outcomes including fractures.^(^ ^13^, ^14^, ^15^, ^16^, ^17^, ^18^, ^19^, ^20^, ^21^, ^22^ ^)^

Existing studies of these biomarkers of vitamin D metabolism and risk of fracture, however, have several limitations. Many examined populations composed of predominantly White, male‐only, or elderly participants with multiple comorbidities.^(^ ^3^, ^4^, ^19^, ^20^, ^21^, ^22^, ^23^, ^24^ ^)^ Results from these studies may not be generalizable to relatively healthy multiethnic populations, given well‐known racial and ethnic differences in vitamin D metabolism.^(^ ^25^ ^)^ Moreover, few studies have accounted for medications with clear implications for fracture risk, such as bisphosphonates and hormone replacement therapy.

We conducted a longitudinal study to test associations of 25(OH)D, the VDMR, PTH, and FGF‐23 with risk of hip and vertebral fractures in a large, multiethnic population of community‐dwelling adults without clinically apparent cardiovascular disease. The work may elucidate the role of existing serum biomarkers of vitamin D metabolism in fracture risk assessment and fracture prevention in relatively healthy multiethnic populations.

## Materials and Methods

### Study population

The Multi‐Ethnic Study of Atherosclerosis (MESA) is an ongoing, community‐based prospective cohort study of cardiovascular disease.^(^ ^26^ ^)^ Between 2000 and 2002, MESA recruited 6814 adults without clinically apparent cardiovascular disease between the ages of 45 and 84 years from six communities located across the United States: Forsyth County, NC; Northern Manhattan and the Bronx, NY; Baltimore and Baltimore County, MD; St. Paul, MN; Chicago, IL; and Los Angeles County, CA. Institutional review boards at all participating centers approved the study, and all participants gave written informed consent.

For this study, we excluded 341 participants without vitamin D measurements at the baseline examination, six participants with 25(OH)D concentrations >100 ng/mL (suggestive of excessive vitamin D supplementation), and one participant with a VDMR of 2000 pg/ng (an extreme outlier), leaving a sample size of 6466.

### Measurements of Vitamin D Metabolism biomarkers

Concentrations of 25(OH)D_2_, 25(OH)D_3_, and 24,25(OH)_2_D_3_ were measured using immunoaffinity extraction and liquid chromatography tandem mass spectrometry (LC–MS/MS) at the University of Washington Nutrition Obesity Research Center from baseline fasting serum samples collected from 2000 to 2002.^(^ ^27^, ^28^, ^29^ ^)^ Calibration of 25(OH)D was confirmed with National Institute of Standards and Technology standard reference material 972a.^(^ ^30^ ^)^ Interassay coefficients of variation calculated using repeat measurements of quality control specimens were 11.8% at 7.0 ng/mL for 25(OH)D_2_; 8.5% at 24.8 ng/mL for 25(OH)D_3_; and 14.7% at 2.7 ng/mL for 24,25(OH)_2_D_3_. Total 25(OH)D was calculated by the sum of measured 25(OH)D_2_ and 25(OH)D_3_ concentrations. Since there was no spectrometric evidence of 24,25(OH)_2_D_2_, the VDMR was calculated by dividing 24,25(OH)_2_D_3_ by 25(OH)D_3_ and then multiplying by 1000, so that its units are in pg/ng.^(^ ^25^, ^31^, ^32^ ^)^ PTH was measured with the Beckman Coulter DxI automated two‐site immunoassay (Beckman Coulter Inc., Brea, CA) and intact FGF‐23 via the Kainos immunoassay (Kainos Laboratories, Tokyo, Japan) using previously unthawed serum.^(^ ^33^, ^34^ ^)^

### Outcomes

The primary outcome was time to a composite of first hip or vertebral fracture. Fracture diagnoses were abstracted from inpatient records through ICD‐9 and ICD‐10 codes. Codes used for hip fracture were ICD‐9: 820.x; ICD‐10: S72.0x, S72.1x, and S72.2x; codes used for vertebral fracture were ICD‐9: 805.x and 806.x; ICD‐10: S22.0x and S32.0x.^(^ ^3^, ^35^, ^36^ ^)^ We used hip fracture codes in any position on a hospital claim and vertebral fracture codes only in the first position on the inpatient claim (i.e., listed as the primary discharge diagnosis) based on a validated algorithm shown to have a positive predictive value for incident fracture that exceeds 95%.^(^ ^35^ ^)^ Additionally, fracture events with concomitant codes for motor vehicle accidents (ICD‐9: E810‐E819; ICD‐10: V89.2) or pathologic fractures (ICD‐9: 733.1x; ICD‐10: M80.x) were excluded. Follow‐up for this study was from the baseline examination through December 31, 2018.

### Covariates

Baseline demographics, including race and ethnicity, smoking status, and comorbidities, were ascertained at the baseline MESA examination through self‐administered questionnaires, interviewer‐administered standardized interviews, extensive in‐person examinations, and laboratory data. Participants identified themselves as belonging to one of four racial or ethnic groups: White, Black, Hispanic, or Chinese. A validated medication inventory was used to assess medication use.^(^ ^37^ ^)^ Diabetes status was defined by the use of an oral hypoglycemic medication or insulin, fasting blood glucose ≥126 mg/dL, nonfasting blood glucose ≥200 mg/dL, or hemoglobin A1c ≥6.5%.^(^ ^38^ ^)^ Weight and height were measured and used to calculate body mass index (BMI) in units of kg/m^2^. Estimated glomerular filtration rate (eGFR) was estimated from serum creatinine using the Chronic Kidney Disease Epidemiology Collaboration equation.^(^ ^39^ ^)^ Serum calcium, serum phosphate, and urine albumin were measured on a Beckman Coulter DxC autoanalyzer (Beckman Coulter Inc.) by indirect potentiometry, timed‐rate colorimetric reaction method, and the modified Doumas and Rodkey procedures, respectively.^(^ ^40^ ^)^ Urine creatinine was measured with the Array 3600 CE Protein Analyzer (Beckman Coulter Inc.) by nephelometry. Albuminuria was quantified as the ratio of albumin to creatinine from a single voided urine sample.

### Statistical analysis

We examined the functional forms of the associations between each biomarker of vitamin D metabolism (25[OH]D, the VDMR, PTH, and FGF‐23) and fracture risk using restricted cubic spline models. We used Cox regression models to assess associations between each biomarker of vitamin D metabolism and time to first hip or vertebral fracture. An initial model was unadjusted, a second model adjusted for age, sex, race and ethnicity, and study site, and a third model additionally adjusted for diabetes, smoking status, systolic blood pressure, BMI, antihypertensive use, statin use, bisphosphonate use, hormone replacement therapy, albuminuria, and eGFR. Adjusted covariates were selected a priori because they could be confounders based on biologic plausibility or because their inclusion would make the primary inference more precise. Interactions were tested using the Wald test of the product terms for each vitamin D metabolism biomarker and race and ethnicity. We performed principal component analysis using serum concentrations of 25(OH)D, PTH, and FGF‐23 and the VDMR as input variables (standardized to a mean of 0 and standard deviation [SD] of 1) to assess proportion of explained variance and whether linear combinations of these biomarkers differed by fracture status. We performed sensitivity analysis to examine hip and all vertebral fractures, including those coded as nonprimary discharge diagnoses, as the outcome. Two‐sided *p* < 0.05 was considered statistically significant. All analyses were conducted with *R* version 3.6.1 (*R* Foundation for Statistical Computing).

## Results

### Participant characteristics

The study cohort was 39% White, 27% Black, 22% Hispanic, and 12% Chinese.

The mean age of participants was 62 ± 10 years, and 53% were female. The mean (SD) VDMR and 25(OH)D were 151.7 (44.4) pg/ng and 25.3 (10.9) ng/mL, respectively. Participants with higher VDMR were more likely to be White, less likely to be Black, less likely to have hypertension or diabetes, had lower concentrations of PTH, and had higher concentrations of 24,25(OH)_2_D_3_, 25(OH)D_3_, and total 25(OH)D (Table 1).

**Table 1:** Characteristics of Participants in MESA by Tertiles of VDMR

	Tertile 1 (*n* = 2156)	Tertile 2 (*n* = 2155)	Tertile 3 (*n* = 2155)
VDMR range (pg/ng)	0–131.91	131.94–167.94	167.96–428.57
Demographics			
Age (years), mean (SD)	63 (10)	62 (10)	62 (10)
Female, *n* (%)	1258 (58)	1116 (52)	1071 (50)
Race, *n* (%)			
White	621 (29)	846 (39)	1040 (48)
Black	822 (38)	543 (25)	395 (18)
Hispanic	484 (22)	485 (23)	442 (21)
Chinese	229 (11)	281 (13)	278 (13)
Study site, *n* (%)			
Forsyth County, NC	336 (16)	291 (14)	344 (16)
Northern Manhattan and the Bronx, NY	376 (17)	345 (16)	293 (14)
Baltimore and Baltimore County, MD	401 (19)	343 (16)	292 (14)
St. Paul, MN	315 (15)	358 (17)	356 (17)
Chicago, IL	349 (16)	379 (18)	414 (19)
Los Angeles County, CA	379 (18)	439 (20)	456 (21)
Medical history and lifestyle			
Hypertension, *n* (%)	1075 (50)	937 (44)	865 (40)
Diabetes, *n* (%)	388 (18)	261 (12)	154 (7)
Smoking status, *n* (%)			
Never	1057 (49)	1114 (52)	1091 (51)
Former	730 (34)	789 (37)	829 (39)
Current	358 (17)	249 (12)	229 (11)
Medication use			
Antihypertensives, *n* (%)	899 (42)	761 (35)	716 (33)
Statins, *n* (%)	321 (15)	320 (15)	324 (15)
Bisphosphonates, *n* (%)	67 (3)	66 (3)	88 (4)
Hormone replacement therapy, *n* (%)	305 (14)	328 (15)	362 (17)
Physical examination data			
BMI (kg/m^2^), mean (SD)	29.7 (6.1)	28.0 (5.1)	27.2 (4.8)
Systolic BP (mmHg), mean (SD)	129 (22)	126 (22)	124 (20)
Diastolic BP (mmHg), mean (SD)	72 (11)	72 (10)	71 (10)
Laboratory data			
Season at blood draw, *n* (%)			
January–March	707 (33)	629 (29)	460 (21)
April–June	679 (32)	631 (29)	643 (30)
July–September	344 (16)	423 (20)	496 (23)
October–December	426 (20)	472 (22)	556 (26)
eGFR (ml/Min per 1.73 m^2^), mean (SD)	76 (18)	78 (16)	79 (14)
UACR (mg/g), median (IQR)	6 (4, 14)	5 (3, 11)	5 (3, 9)
Calcium (mg/dL), mean (SD)	9.6 (0.4)	9.6 (0.4)	9.7 (0.4)
Phosphate (mg/dL), mean (SD)	3.7 (0.5)	3.7 (0.5)	3.7 (0.5)
FGF‐23 (pg/mL), median (IQR)	36 (29, 45)	38 (31, 46)	39 (32, 48)
PTH (pg/mL), median (IQR)	47 (36, 62)	40 (31, 52)	36 (28, 46)
24,25(OH)_2_D_3_ (ng/mL), mean (SD)	1.9 (1.1)	3.5 (1.5)	5.6 (2.6)
25(OH)D_3_ (ng/mL), mean (SD)	17.0 (8.3)	23.1 (9.4)	27.9 (11.0)
Total 25(OH)D (ng/mL), mean (SD)	19.1 (9.2)	26.0 (9.7)	30.8 (10.4)

Abbreviations: 24,25(OH)_2_D_3_, 24,25‐dihydroxyvitamin D_3_; 25(OH)D, 25‐hydroxyvitamin; BMI, body mass index; BP, blood pressure; eGFR, estimated glomerular filtration rate; FGF‐23, fibroblast growth factor‐23; IQR, interquartile range; MESA, Multi‐Ethnic Study of Atherosclerosis; PTH, parathyroid hormone; SD, standard deviation; UACR, urine albumin to creatinine ratio; VDMR, vitamin D metabolite ratio.

### Fracture events

We observed 128 hip (104) and vertebral^(^ ^24^ ^)^ fractures over a mean follow‐up of 14.2 ± 4.9 years for an incidence rate of 1.4 events per 1000 person‐years. The incidence rate among men was 1.1 events per 1000 person‐years, and among women it was 1.7 events per 1000 person‐years.

### Vitamin D metabolism biomarkers and fracture risk

**Fig. 1:**
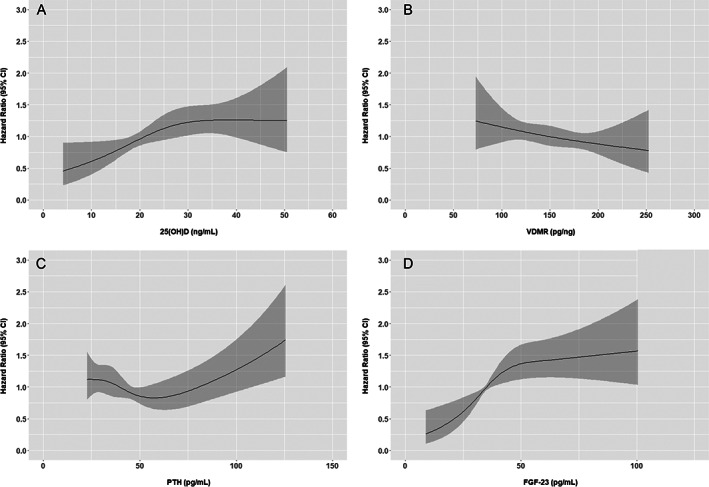
Associations between vitamin D metabolism biomarkers as continuous variables and risk of hip and vertebral fractures. The restricted cubic spline regression models estimate the hazard ratio of hip and vertebral fractures according to (*A*) 25(OH)D, (*B*) VDMR, (*C*) PTH, and (*D*) FGF‐23. The shaded areas represent the 95% confidence interval. CI, confidence interval; 25(OH)D, 25‐hydroxyvitamin D; VDMR, vitamin D metabolite ratio; PTH, parathyroid hormone; FGF‐23, fibroblast growth factor‐23.

**Fig. 2:**
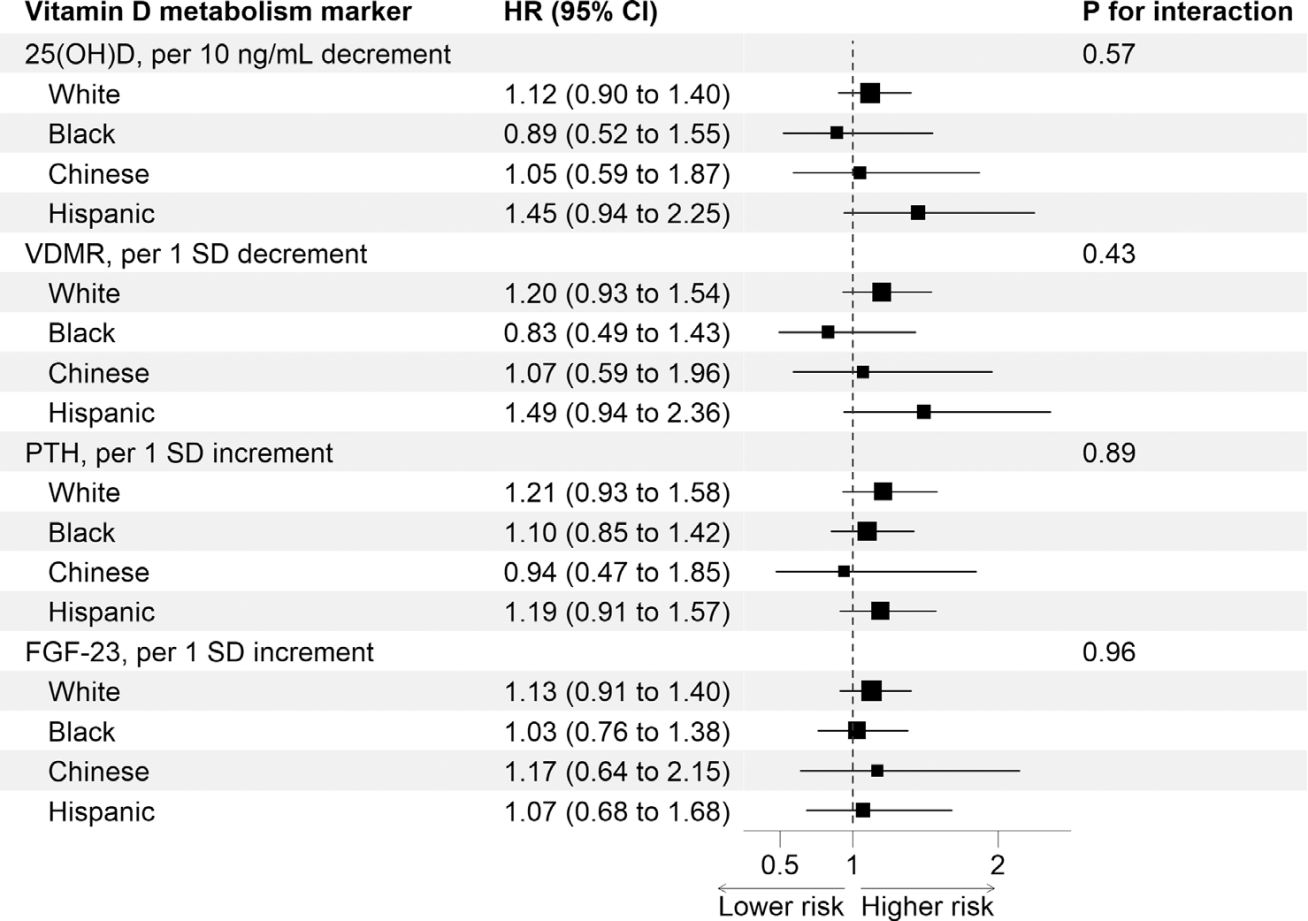
Associations of vitamin D metabolism biomarkers with hip and vertebral fractures by race and ethnicity. The SD for VDMR, PTH, and FGF‐23 are 44 pg/ng, 22 pg/mL, and 18 pg/mL, respectively. *p*‐value for interaction uses White participants as the reference group. Hazard ratio is adjusted for age, sex, study site, diabetes, smoking status, systolic blood pressure, body mass index, medication use (antihypertensives, statins, bisphosphonates, hormone replacement therapy), albuminuria, and estimated glomerular filtration rate. HR, hazard ratio; CI, confidence interval; 25(OH)D, 25‐hydroxyvitamin D; VDMR, vitamin D metabolite ratio; SD, standard deviation; PTH, parathyroid hormone; FGF‐23, fibroblast growth‐factor 23.

Restricted cubic splines suggested roughly linear unadjusted associations of each biomarker of vitamin D metabolism with fracture risk, positive for 25(OH)D, PTH, and FGF‐23, and inverse for the VDMR (Fig. 1). The association of PTH with fracture appeared potentially nonlinear, but a Wald test of the quadratic term was insignificant (*p* = 0.11). In unadjusted models, 25(OH)D was associated with fracture risk, although this association was no longer statistically significant in the adjusted models (hazard ratio [HR] 1.18 per 10 ng/mL lower 25[OH]D; 95% confidence interval [CI]: 0.94, 1.49; Table 2). Neither the VDMR nor PTH was associated with fracture risk in unadjusted or adjusted models. In unadjusted models, FGF‐23 was associated with fracture risk, but this association was no longer statistically significant in the adjusted models (HR 1.02 for each SD higher FGF‐23; 95% CI: 0.79, 1.33). We did not observe significant heterogeneity in the association of any biomarker of vitamin D metabolism and fracture risk by race and ethnicity, with *p*‐values for interaction ≥0.21 across all racial and ethnic groups after full covariate adjustment (Fig. 2). There was also no significant interaction between any of the vitamin D biomarkers and sex. In sensitivity analyses that used hip and all vertebral fractures coded as primary and nonprimary discharge diagnoses as the outcome, results were similar to that of the primary analysis (Table S1).

**Table 2:** Associations of Vitamin D Metabolism Biomarkers with Hip and Vertebral Fracture

	*N* fractures/*N* at risk	Incidence rate (events/1000 person‐years)	Hazard ratio (95% CI)		
			Model 1	Model 2	Model 3
25(OH)D (ng/mL)					
< 20	32/2214	1.0	0.59* (0.38, 0.91)	1.17 (0.73, 1.87)	1.50 (0.82, 2.68)
20 to <30	42/2144	1.4	0.80 (0.53, 1.20)	1.08 (0.72, 1.62)	1.04 (0.59, 1.83)
≥ 30	54/2108	1.8	Ref	Ref	Ref
Per 10 ng/mL decrement			0.84* (0.72, 0.98)	1.06 (0.90, 1.27)	1.18 (0.94, 1.49)
VDMR					
Tertile 1	48/2156	1.6	1.44 (0.94, 2.23)	1.60* (1.03, 2.47)	1.58 (0.90, 2.77)
Tertile 2	44/2155	1.4	1.25 (0.80, 1.94)	1.25 (0.81, 1.95)	0.89 (0.49, 1.61)
Tertile 3	36/2155	1.1	Ref	Ref	Ref
Per 1 SD decrement			1.12 (0.94, 1.34)	1.19 (0.98, 1.44)	1.16 (0.91, 1.48)
PTH (pg/mL)					
< 33	46/1914	1.6	Ref	Ref	Ref
33–65	66/3768	1.2	0.75 (0.52, 1.10)	0.70 (0.48, 1.03)	0.82 (0.50, 1.35)
> 65	16/772	1.6	0.97 (0.55, 1.71)	0.97 (0.54, 1.75)	0.92 (0.40, 2.12)
Per 1 SD increment			1.13 (0.96, 1.31)	1.11 (0.96, 1.32)	1.04 (0.81, 1.33)
FGF‐23					
Tertile 1	33/2152	1.1	Ref	Ref	Ref
Tertile 2	42/2151	1.4	1.28 (0.81, 2.01)	1.08 (0.68, 1.71)	0.84 (0.47, 1.50)
Tertile 3	53/2151	1.8	1.67* (1.08, 2.58)	1.16 (0.74, 1.81)	0.93 (0.52, 1.66)
Per 1 SD increment			1.11* (1.03, 1.20)	1.11 (0.98, 1.26)	1.02 (0.79, 1.33)

*Note*: The SDs for VDMR, PTH, and FGF‐23 are 44 pg/ng, 22 pg/mL, and 18 pg/mL, respectively. Model 1 is unadjusted. Model 2 adjusted for age, sex, race and ethnicity, and study site. Model 3 additionally adjusted for diabetes, smoking status, systolic blood pressure, body mass index, medication use (antihypertensives, statins, bisphosphonates, hormone replacement therapy), albuminuria, and estimated glomerular filtration rate.

Abbreviations: 25(OH)D, 25‐hydroxyvitamin D; CI, confidence interval; FGF‐23, fibroblast growth‐factor 23; PTH, parathyroid hormone; SD, standard deviation; VDMR, vitamin D metabolite ratio.

* *p* < 0.05 compared with the reference group.

**Fig. 3:**
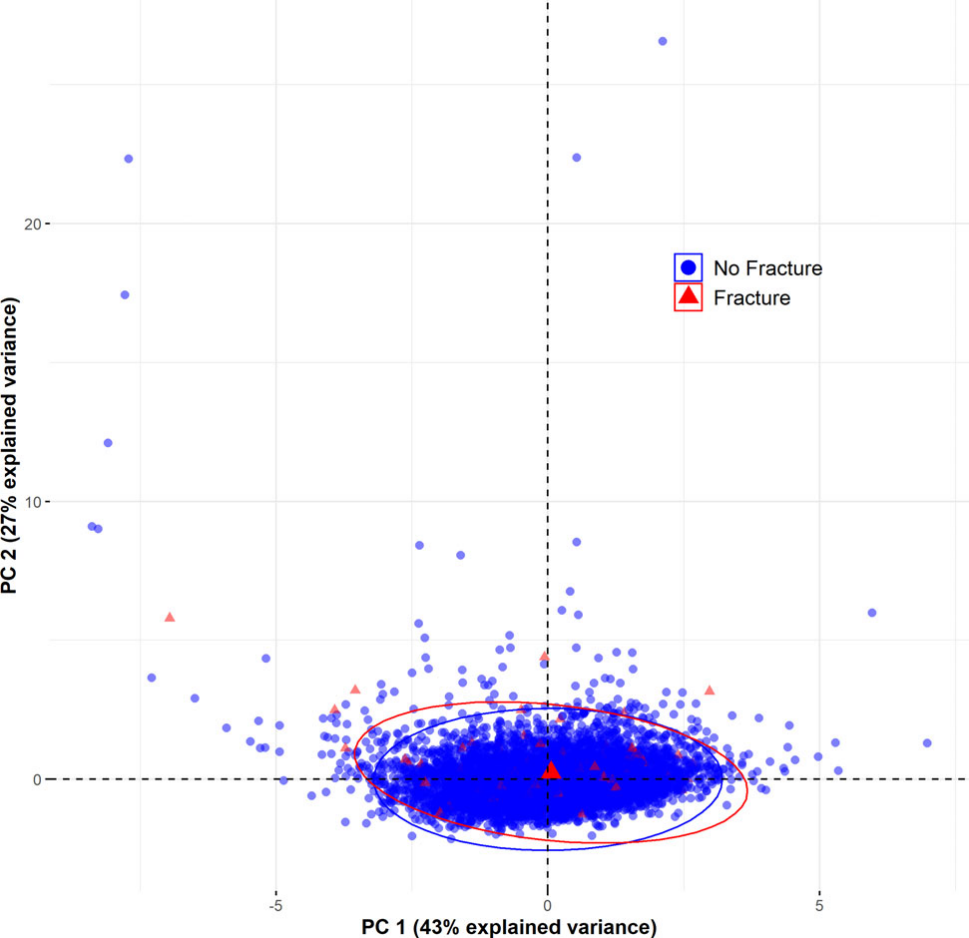
Principal component analysis of vitamin D metabolism biomarkers by fracture status. Principal components (PCs) were constructed as linear combinations of serum concentrations of 25(OH)D, PTH, and FGF‐23 and the VDMR. PC 1 and PC 2 explain 43% and 27% of the total variance in vitamin D metabolism markers, respectively, and cumulatively explain 70% of the total variance. The concentration ellipses assume multivariate normality and enclose 95% of data for each group. The value of PC 1 did not differ significantly between participants who experienced fractures and those who did not (*p* = 0.66). 25(OH)D, 25‐hydroxyvitamin D; VDMR, vitamin D metabolite ratio; PTH, parathyroid hormone; FGF‐23, fibroblast growth factor‐23.

Principal component analysis showed that the first principal component explained 43% of the total variance in vitamin D metabolism biomarkers and was not statistically different between participants who experienced fractures and those who did not (*p* = 0.66; Fig. 3).

## Discussion

In a large, community‐based, multiethnic cohort of adults, we assessed associations between biomarkers of vitamin D metabolism and risk of hip and vertebral fractures. 25(OH)D, VDMR, PTH, and FGF‐23 were not independently associated with fracture risk, and linear combinations of these biomarkers did not differ between participants who experienced fractures and those who did not. Moreover, results were similar irrespective of race or ethnicity.

Racial and ethnic differences in circulating vitamin D metabolites attributable to genetic ancestry are well described,^(^ ^25^ ^)^ and associations between these metabolites and outcomes differ by race.^(^ ^41^, ^42^, ^43^, ^44^ ^)^ As such, studies of vitamin D biomarkers in diverse populations are imperative. A paradigmatic example of this importance is found through the vitamin D paradox,^(^ ^45^ ^)^ where Black populations have a lower incidence of osteopenia and fractures than White populations despite lower concentrations of 25(OH)D, higher PTH, and lower VDMR, all thought to indicate reduced vitamin D sufficiency and activity. Moreover, although studies in elderly, predominantly White populations have suggested low 25(OH)D and high PTH as risk factors for low BMD and fracture risk,^(^ ^3^, ^4^, ^13^, ^14^, ^15^, ^16^, ^23^, ^24^ ^)^ a case–control study by Cauley et al.—among the few to examine a multiethnic population—showed that associations of lower 25(OH)D with fracture risk in White women do not hold true for Black, Hispanic, Asian, or American Indian women.^(^ ^44^ ^)^ MESA presents a unique and important cohort to test whether associations of biomarkers of vitamin D metabolism and fracture risk extend to relatively younger and healthy, multiethnic populations of men and women, and we found that they did not.

Although 1,25(OH)_2_D is the active vitamin D metabolite, its concentrations do not reflect vitamin D status and may be normal or paradoxically increased in vitamin D deficiency as a result of compensatory mechanisms.^(^ ^46^ ^)^ The circulating concentration of 25(OH)D is the most widely used measure of vitamin D status but merely reflects vitamin D exposure and provides limited information on tissue‐level vitamin D activity and bone health. Indeed, even at 25(OH)D concentrations less than 10 ng/mL—far below the 20–30 ng/mL experts consider to be sufficient—greater than 50% of participants in the study by Priemel et al. had no biopsy evidence of osteomalacia.^(^ ^6^ ^)^ Unsurprisingly, then, associations between 25(OH)D and fractures have been inconsistent. In two elderly populations with limited ethnic representation, we previously observed no association between 25(OH)D and risk of hip or all fractures.^(^ ^3^, ^4^ ^)^ Here we confirm and extend these findings to a large, relatively younger multiethnic cohort with available long‐term follow‐up.

The VDMR has been used as a measure of functional vitamin D activity and validated as a surrogate measure of 25(OH)D_3_ clearance, a process driven predominantly by vitamin D receptor activation by 1,25(OH)_2_D.^(^ ^47^ ^)^ We previously examined the VDMR–fracture relationship in two elderly populations and found lower VDMR was associated with increased fracture risk, whereas 25(OH)D was not in the same individuals.^(^ ^3^, ^4^ ^)^ As elaborated earlier, differences between study populations may explain the conflicting results between those studies and the present one. Additionally, our two prior studies lacked data on bisphosphonate use or hormone replacement therapy, unlike this study, and may be confounded.

Nephrologists commonly use PTH as a biomarker of bone turnover in chronic kidney disease (CKD), and studies consistently show associations between PTH and fractures in patients with CKD.^(^ ^48^, ^49^, ^50^, ^51^ ^)^ Studies examining the relationship between PTH and fractures in populations with normal kidney function are much more sparse, and those that have examined the PTH–bone relationship have largely used BMD as an outcome. In these studies, when involving majority White and European populations, PTH has been consistently inversely associated with BMD.^(^ ^13^, ^14^, ^15^, ^16^ ^)^ However, in more racially and ethnically diverse cohorts, associations between PTH and BMD are either attenuated or entirely absent.^(^ ^17^, ^18^ ^)^ The lack of an association between PTH and fracture risk in our multiethnic cohort appears consistent with the BMD studies in multiethnic cohorts by others.

As the newest of the four biomarkers tested in this study, FGF‐23 has received intense interest as a potential biomarker of bone health since its discovery in 2003. Several studies have examined the relationship of FGF‐23 with fracture risk and, consistently with our study, have found no significant association between them.^(^ ^19^, ^20^, ^21^ ^)^ The one exception is a study by Mirza et al., which linked higher FGF‐23 concentrations with higher fracture risk in a cohort of elderly Swedish men.^(^ ^22^ ^)^ Fracture rates in Sweden are among the highest in the world^(^ ^52^ ^)^—3.6 hip and 6.4 vertebral fractures per 1000 person‐years in the Mirza study compared with 1.1 composite hip and vertebral fractures per 1000 person‐years in men in our study of community‐dwelling Americans. Genetic, socioeconomic, or environmental differences between the two study populations may explain the contrasting findings. Altogether, it seems unlikely that FGF‐23 is a risk factor for fractures in most community‐dwelling adult men and women.

Findings from observational studies like the current study need to be supported by interventional trials. Indeed, LeBoff et al. recently published a randomized trial that showed no effect of vitamin D supplementation on incident fractures,^(^ ^53^ ^)^ adding to the growing number of trials showing a lack of beneficial effects of vitamin D on clinical outcomes.^(^ ^53^, ^54^, ^55^, ^56^, ^57^ ^)^ It remains important to note that these studies were conducted in vitamin D–replete members of the general population, which are poorly representative of populations like those with CKD and kidney failure who experience significant disturbances in vitamin D metabolism. Studies that proactively address best practices in populations such as CKD and kidney failure are even more necessary to prevent the inappropriate generalization of results from vitamin D studies conducted in the general population.

Along these lines, we emphasize that our results reflect a study performed in a relatively healthy, younger, largely vitamin D–replete, multiethnic population in whom the risk of fracture is likely low. Vitamin D deficiency and secondary hyperparathyroidism are very common in geriatric patients, nearly universal in the institutionalized elderly,^(^ ^58^, ^59^, ^60^ ^)^ and more consistently associated with falls, bone loss, and later fractures in these groups than the general population.^(^ ^60^, ^61^, ^62^, ^63^ ^)^ Furthermore, clinical trials show vitamin D supplementation with calcium reduce fall and fracture risk in institutionalized patients and elderly people living at home.^(^ ^63^, ^64^, ^65^, ^66^, ^67^ ^)^ Thus, biomarkers of vitamin D metabolism may be differentially associated with fracture risk in other populations, such as the institutionalized elderly.

To our knowledge, our study of adult men and women is among the largest and most racially and ethnically diverse to explore associations of vitamin D metabolism biomarkers and fractures. Other strengths include accurate measurements of vitamin D metabolites using LC–MS/MS and consistent results in sensitivity analyses. This study also has important limitations. First, although our study used a validated ICD‐based algorithm with >95% positive predictive value for hip and vertebral fractures,^(^ ^35^ ^)^ our reliance on hospital discharge data meant that outpatient fractures were missed. Vertebral fractures in particular were likely underdiagnosed, given that they generally occur outside of the hospital and occurred at a rate several fold lower in this cohort than expected relative to hip fractures based on other epidemiologic studies. The impact of missing outpatient fractures would appear to be an attenuation of any true associations between biomarkers of vitamin D metabolism and fracture risk, given the lower event rate and the concept that nondifferential misclassifications of outcomes tend to bias associations toward the null. Second, we lacked data on fracture prevalence and could not determine whether fractures during the study period were truly incident, which increases risk of confounding and reverse causation. Third, although we did not find significant interaction between any of the biomarkers and race and ethnicity, our study was likely underpowered to detect associations at the subgroup level. Fourth, we recognize race and ethnicity as social constructs and weak proxies for biological and genetic diversity. Misclassifications in genetic ancestry may have occurred and also diluted our ability to detect interactions by race and ethnicity. Fifth, our exposures were measured only once, although 25(OH)D concentrations have been shown to be stable over time within individuals in MESA.^(^ ^27^ ^)^ Lastly, we lacked data on vitamin D supplementation and could not adjust for it in our analyses.

In conclusion, 25(OH)D, VDMR, PTH, FGF‐23, or combinations of these biomarkers of vitamin D metabolism do not appear to be risk factors for fractures in community‐dwelling, relatively healthy, multiethnic adults with normal kidney function. Actionable biomarkers associated with fractures in this population are needed to guide evaluation and treatment to prevent bone disease.

## AUTHOR CONTRIBUTIONS

**Simon Hsu:** Conceptualization; formal analysis; funding acquisition; investigation; methodology; resources; visualization; writing – original draft; writing – review and editing. **Michael Criqui:** Funding acquisition; investigation; resources; writing – review and editing. **Charles Ginsberg:** Funding acquisition; investigation; resources; writing – review and editing. **Andrew Norbert Hoofnagle:** Funding acquisition; investigation; resources; writing – review and editing. **Joachim H. Ix:** Funding acquisition; investigation; resources; writing – review and editing. **Robyn L. McClelland:** Funding acquisition; investigation; resources; writing – review and editing. **Erin D. Michos:** Funding acquisition; investigation; resources; supervision; writing – review and editing. **Steven J. Shea:** Investigation; methodology; resources; supervision; writing – review and editing. **David Siscovick:** Funding acquisition; investigation; resources; supervision; writing – review and editing. **Leila Zelnick:** Formal analysis; funding acquisition; investigation; methodology; resources; visualization; writing – review and editing. **Bryan Kestenbaum:** Funding acquisition; investigation; resources; visualization; writing – review and editing. **Ian H. de Boer:** Conceptualization; funding acquisition; investigation; methodology; resources; supervision; visualization; writing – original draft; writing – review and editing.

## AUTHORS' ROLES

Conceptualization: S.H. and I.H.d.B. Methodology: S.H., I.H.d.B., and L.R.Z. Formal analysis: S.H. and L.R.Z. Investigation, all authors. Resources, all authors. Writing—original draft: S.H. and I.H.d.B. Writing—review and editing: all authors. Visualization: S.H., I.H.d.B., L.R.Z., and B.R.K. Supervision—E.D.M., S.J.S., D.S., and I.H.d.B. Funding acquisition—all authors.

### PEER REVIEW

The peer review history for this article is available at https://publons.com/publon/10.1002/jbm4.10697.

## Supporting information


**Table S1.** Associations of vitamin D metabolism biomarkers with hip and vertebral fracture, including all vertebral fractures coded as primary and non‐primary discharge diagnoses.
