# Supplementary material for: Biomarkers of Vitamin D Metabolism and Hip and Vertebral Fracture Risk: The Multi‐Ethnic Study of Atherosclerosis
Source: JBMR Plus. 2022 Nov 11;6(12):e10697. doi: 10.1002/jbm4.10697 (PMC9751658; doi:10.1002/jbm4.10697)
Supplement: Supplementary file 1 — Table S1. Associations of vitamin D metabolism biomarkers with hip and vertebral fracture, including all vertebral fractures coded as primary and non‐primary discharge diagnoses. [file JBM4-6-e10697-s001.docx]

**Supplementary Table 1.** Associations of vitamin D metabolism biomarkers with hip and vertebral fracture, including all vertebral fractures coded as primary and non-primary discharge diagnoses

|  | **N fractures / N at risk** | **Incidence rate (events/1000 person-years)** | **Hazard Ratio (95% CI)** | | |
| --- | --- | --- | --- | --- | --- |
|  |  |  | **Model 1** | **Model 2** | **Model 3** |
| **25(OH)D (ng/mL)** |  |  |  |  |  |
| <20 | 34 / 2214 | 1.1 | 0.56* (0.37, 0.85) | 1.10 (0.70, 1.72) | 1.33 (0.74, 2.39) |
| 20 to <30 | 49 / 2144 | 1.6 | 0.84 (0.58, 1.23) | 1.12 (0.77, 1.65) | 1.07 (0.63, 1.83) |
| >30 | 60 / 2108 | 2.0 | Ref | Ref | Ref |
| Per 10 ng/mL decrement |  |  | 0.83* (0.72, 0.95) | 1.04 (0.88, 1.23) | 1.13 (0.91, 1.41) |
| **VDMR** |  |  |  |  |  |
| Tertile 1 | 49 / 2156 | 1.7 | 1.27 (0.84, 1.91) | 1.39 (0.92, 2.11) | 1.34 (0.78, 2.29) |
| Tertile 2 | 52 / 2155 | 1.7 | 1.26 (0.84, 1.90) | 1.28 (0.85, 1.93) | 0.87 (0.50, 1.51) |
| Tertile 3 | 42 / 2155 | 1.3 | Ref | Ref | Ref |
| Per 1 SD decrement |  |  | 1.05 (0.89, 1.24) | 1.10 (0.92, 1.32) | 1.06 (0.84, 1.34) |
| **PTH (pg/mL)** |  |  |  |  |  |
| <33 | 51 / 1914 | 1.8 | Ref | Ref | Ref |
| 33-65 | 74 / 3768 | 1.4 | 0.76 (0.53, 1.09) | 0.71 (0.50, 1.03) | 0.74 (0.46, 1.19) |
| >65 | 18 / 772 | 1.7 | 0.98 (0.57, 1.68) | 1.00 (0.57, 1.74) | 1.03 (0.48, 2.18) |
| Per 1 SD increment |  |  | 1.11 (0.96, 1.29) | 1.11 (0.96, 1.30) | 1.06 (0.83, 1.34) |
| **FGF-23** |  |  |  |  |  |
| Tertile 1 | 34 / 2152 | 1.1 | Ref | Ref | Ref |
| Tertile 2 | 49 / 2151 | 1.6 | 1.45 (0.93, 2.24) | 1.23 (0.79, 1.91) | 0.95 (0.54, 1.67) |
| Tertile 3 | 60 / 2151 | 2.0 | 1.84* (1.21, 2.80) | 1.29 (0.84, 1.98) | 1.08 (0.62, 1.90) |
| Per 1 SD increment |  |  | 1.11* (1.04, 1.19) | 1.11 (0.98, 1.24) | 1.06 (0.84, 1.34) |

When including all hip and vertebral fractures regardless of diagnosis code position in the outcome, we observed 143 hip (104) and vertebral (39) fractures over mean (SD) follow-up of 14.2 (4.9) years for an incidence rate of 1.6 fractures per 1000 person-years. The SD for VDMR, PTH, and FGF-23 are 44 pg/ng, 22 pg/mL, and 18 pg/mL, respectively. Model 1 is unadjusted. Model 2 adjusted for age, sex, race and ethnicity, and study site. Model 3 additionally adjusted for diabetes, smoking status, systolic blood pressure, body mass index, medication use (anti-hypertensives, statins, bisphosphonates, hormone replacement therapy), albuminuria, and estimated glomerular filtration rate.

*P<0.05 compared with the reference group.

CI, confidence interval; 25(OH)D, 25-hydroxyvitamin D; VDMR, vitamin D metabolite ratio; SD, standard deviation; PTH, parathyroid hormone; FGF-23, fibroblast growth-factor 23
